# Epidemic area contact history and sleep quality associated with posttraumatic stress symptoms in the first phase of COVID-19 outbreak in China

**DOI:** 10.1038/s41598-020-80649-8

**Published:** 2020-12-31

**Authors:** Fan Zhang, Zhilei Shang, Haiying Ma, Yanpu Jia, Luna Sun, Xin Guo, Lili Wu, Zhuoer Sun, Yaoguang Zhou, Yan Wang, Nianqi Liu, Weizhi Liu

**Affiliations:** 1 Lab for Post-Traumatic Stress Disorder, Faculty of Psychology and Mental Health, Naval Medical University, #800 Xiangyin Road, Shanghai, 200433 China; 2 The Emotion & Cognition Lab, Faculty of Psychology and Mental Health, Naval Medical University, Shanghai, 200433 China; 3 Counselling and Psychological Services Center, Faculty of Psychology and Mental Health, Naval Medical University, Shanghai, 200433 China; 4 The Battalion 5 of Cadet Brigade, School of Basic Medicine, Naval Medical University, Shanghai, 200433 China

**Keywords:** Psychology, Diseases

## Abstract

The impact of 2019 coronavirus disease (COVID-19) outbreak on mental health was of widespread concern recently. The present study aimed to exam sleep quality and posttraumatic stress symptoms (PTSS) and potential influence factors in the first phases of COVID-19 massive outbreak in China. A snowball sampling technique was used and a total of 2027 Chinese participated in the present study. Demographic information, epidemic area contact history, sleep quality and PTSS data were collected with an internet-based cross-sectional survey. Results suggested that 59.7% participants were not fully satisfied with their sleep quality, and 50.9% participants had various degrees of short sleep duration problems. 44.1% and 33.0% participants had sleep disturbance and sleep onset latency problems. Also, the prevalence of PTSS reached 4.7% in the self-rating survey. Epidemic area contact history affected PTSS and latency onset of sleep under the influence of COVID-19. Epidemic area contact history and sleep quality had interaction effects on PTSS. The present study was one of the first to evaluate acute psychological responses and possible risk factors during the peak of COVID-19 in China and results indicate that keeping good sleep quality in individuals with pandemic exposure experiences is a way to prevent PTSS.

## Introduction

According to the Diagnostic and Statistical Manual of Mental Disorders-5th edition (DSM-5)^1^, Posttraumatic Stress Disorder (PTSD) is characterized by four symptom groups: (a) involuntary memories of the trauma such as intrusions or nightmares; (b) persistent avoidance of stimuli associated with the traumatic event; (c) negative alterations in cognitions and mood that are associated with the trauma; and (d) alterations in arousal and reactivity that are associated with the trauma. Exposure to traumatic events is the immediate cause of PTSD and an essential condition of diagnosis. Being infected with a fatal epidemic disease might be a life-threating event in some patients and could be a cause of posttraumatic stress symptoms. For example, 12%-15% survivors of Severe Acute Respiratory Syndrome (SARS), a severe infectious disease, showed one type of PTSD symptoms (PTSS) one month after discharge^2^; Keita et al. showed in a small-sample study that the outbreak of Ebola virus disease (EVD) in West Africa caused 9.1% morbidity of PTSD in EVD survivors^3^. In addition, not only being patients could develop PTSS, living in epidemic area and receiving disease-related information would also arouse risk perception and lead to PTSS like irrational nervousness or scare^4^. Recently, Forte et al.^5^ showed that the COVID-19 pandemic caused various PTSD symptoms and could be considered as a traumatic event. Additionally, to control the transmission of epidemics, large numbers of people were placed into quarantine, which was identified as a risk factor of PTSS^6,7^.

**Figure 1:**
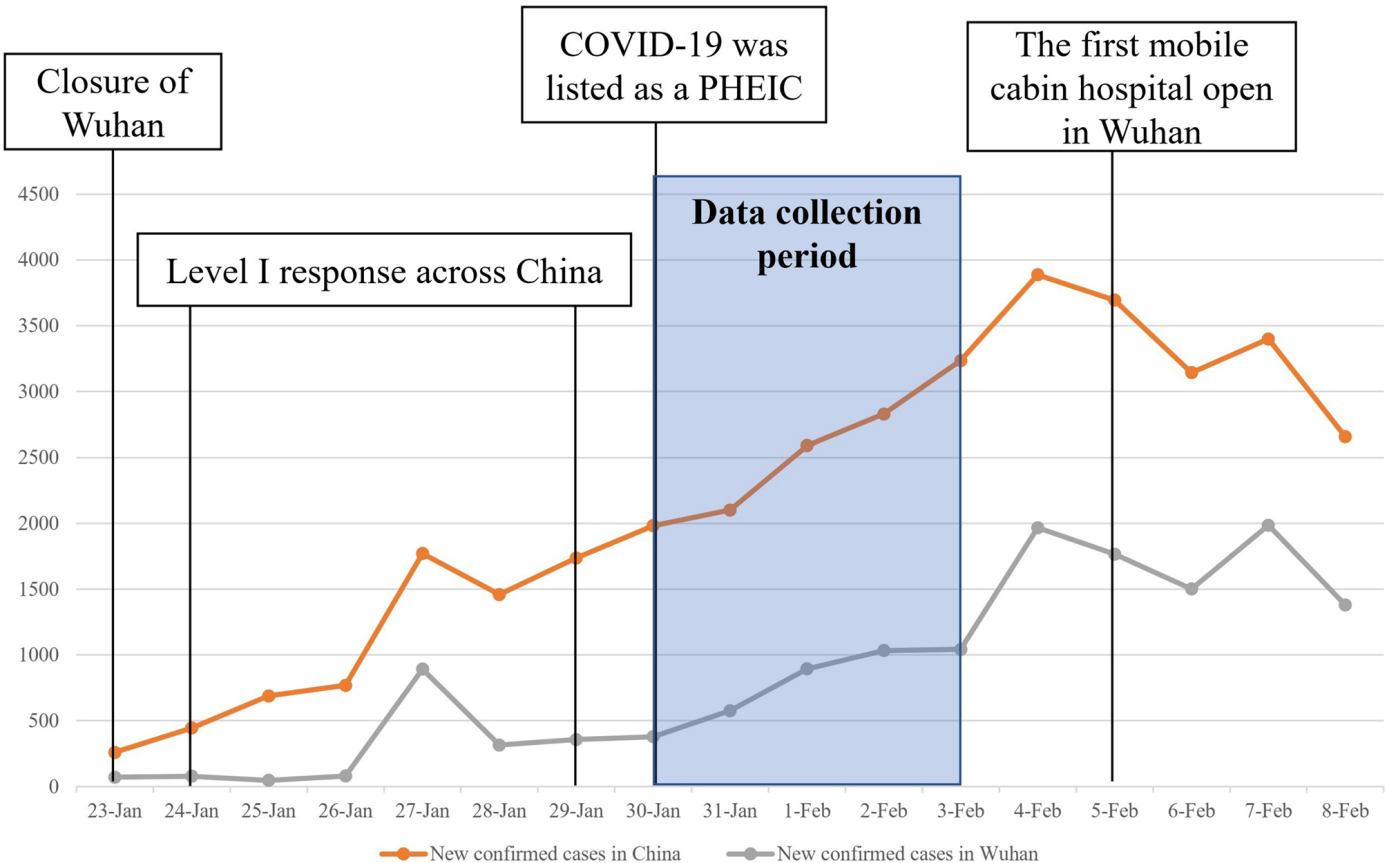
New confirmed cases in China and Wuhan during data collection period. Data were from National Health Commission of China and Health Commission of Hubei Providence. *PHEIC *the Public Health Emergency of International Concern.

The 2019 Coronavirus disease (COVID-19) outbreak in China was an unprecedented, large-scale public health emergency causing substantial human suffering and deaths. On Jan 30, 2020 the outbreak of COVID-19 has been determined by the World Health Organization WHO as a Public Health Emergency of International Concern (PHEIC)^8^. From the end of January to the beginning of February, the new confirmed cases and accumulated confirmed cases grew rapidly without showing any sign of decrease (see Fig. 1). By the end of the study (up to Feb 4, 2020), COVID-19 had caused 20,471 confirmed cases and 425 deaths in China^9^. Although having experienced the outbreak of SARS which had caused hundreds of deaths in China in 2003^10^, the rapid spread of infection and the characteristic of human transmission of COVID-19^11^ were beyond expectation, causing anxiety, depression and PTSD throughout the world^12–14^. However, PTSS, the psychological responses which might be closely linked with life-threatening event, had received less attention during the peak of infection and death of COVID-19 in China. Hence in the present study, we focused on the acute PTSS in Chinese under the background of massive COVID-19 outbreak.

There is currently plenty of evidence to suggest that poor sleep quality is closely related to PTSD. For instance, a study of 2627 American adults illustrated that 92% individuals who met DSM-5 criteria for PTSD showed at least one sleep disturbance like insomnia and nightmare^15^. Sleep has also been shown to be related with the development and severity of PTSD. For example, researches have shown that subjective insomnia, nightmare, and fragmentation of REM sleep in the early aftermath of a traumatic event could predict later development of PTSD^16,17^. Casement et al.^18^ showed that the severity of PTSD in female PTSD patients was associated with the severity of sleep quality problems. Sleep disturbance might affect fear extinction, which is a key element of recovery from trauma^19^ and poor sleep could affect adaptive emotion regulation^20^. These might be possible mechanisms underlying the connection between sleep and PTSD. Also, interventions on sleep quality could relieve PTSD, indicating the impact of sleep in the course and treatment of PTSD^21^. In brief, sleep might play an important role in the process of PTSD. However, to keep high-quality sleep is not as easy as it seems. Previous studies have proved that massive outbreak of epidemics like SARS and EVD could cause sleep problems in relevant individuals. For example, Yu et al.^22^ reported that midlife women felt “sleep was restless” during SARS outbreak and this problem was closely associated with their overall negative emotions. Another study showed that chronic post-SARS symptoms were characterized by sleep-related problems^23^. Survivors of EVD also reported various sleep quality problems^3^. A recent study investigating 2291 Italian residents during massive outbreak of COVID-19 in Italia reported that more than half of the subjects showed poor sleep quality^24^. The prevalence of COVID-19-related sleep quality problems in Chinese general population were much lower than that in Italians, but a 18.2% occurrence rate was still alarming^14^. Taking the association between sleep quality and PTSD into consideration, the present study aimed to investigate the influence of COVID-19 on sleep quality and further to explore how the influence of COVID-19 and sleep quality problems would affect PTSS.

In early December 2019, the first pneumonia case was identified in Wuhan, the capital city of Hubei province^25^. With the spreading of COVID-19, Wuhan became the worst influenced area, where the mortality and morbidity of COVID-19 were above the national level according to the National Health Commission of China (NHC)^26^. Moreover, 72.3% confirmed cases were not residents of Wuhan but had contact with individuals lived in Wuhan^27^. Thus, Wuhan expose history had become an important diagnosis standard^28^ and a source of fear in the first phase of COVID-19 in China. As a result, data of Wuhan exposure history was collected to measure the risk of infection. A previous study had showed that health care workers who worked in high-risk locations or had friends or close relatives who had been exposed to SARS were 2–3 times more likely to have high PTSS^29^, indicating that high risks of infection may aggravate PTSS severity. Also, high infectious risk was related with poor sleep in health care workers^30^. According to these existing evidences, it was supposed that individuals who had contact with epidemic area like Wuhan might show more PTSS.

Taken together, the present study aimed to investigate PTSS in general population in China during early massive outbreak of COVID-19. Data on sleep quality and history of epidemic area contact were collected to analyze the risk factor of PTSS during COVID-19. The hypotheses were: (1) general population would show various sleep quality problems and PTSS under the influence of COVID-19; (2) Wuhan residents and individuals who had been to Wuhan or contacted with Wuhan residents, would show more sleep problems and PTSS for they were faced with higher possibility of infection; (3) poorer sleep quality would be related with more PTSS and sleep quality and might have joint effects with Wuhan contact history on PTSS. The current study could reflect the influence of COVID-19 on psychological well-being in Chinese and shed light on post-epidemic psychological intervention in China and around the world.

## Methods

The methods and reporting procedures were in accordance with the STROBE (Strengthening the Reporting of Observational studies in Epidemiology) checklist.

### Participants

**Figure 2:**
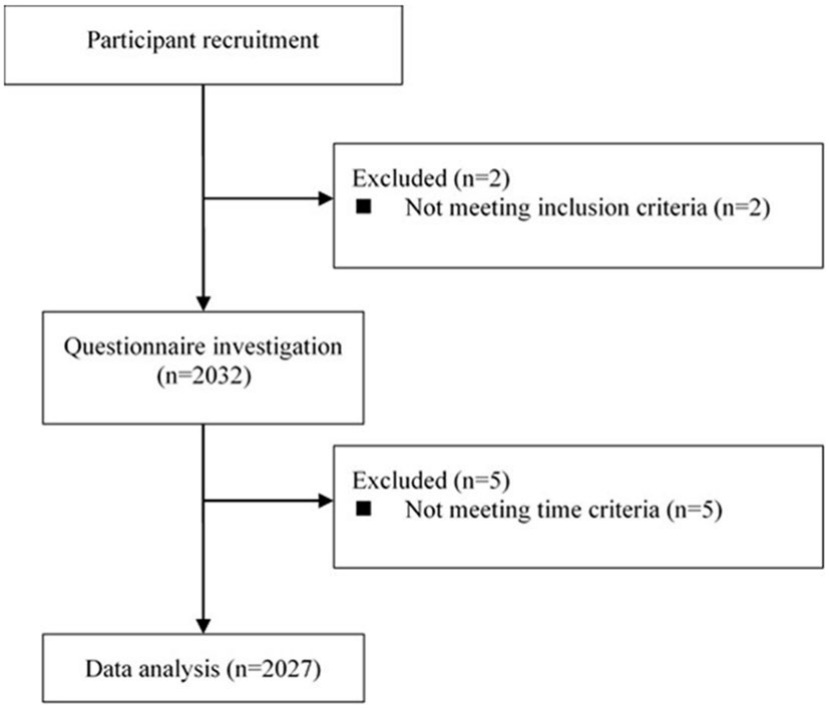
Flowchart depicting passage of participants.

The study began one month after WHO China Office was informed of cases of the unknown etiology detected in Wuhan City of China^31^. A web-based cross-sectional survey, implemented using the Questionnaire Star platform (https://www.wjx.cn) and broadcasted through mainstream social-media Wechat was used to collect data in the Chinese population. And a snowball sampling method was applied to invite potential study participants. The survey was enabled from Jan 30th to Feb 3rd (see Fig. 1). A total of 2032 subjects participated in the present study (5 were deleted because of invalid answer time). Referring to the previous study^32^, criteria for inclusion were that subjects should be Chinese race and age ≥ 18 years at the time of the COVID-19 infection. Figure 2 demonstrated passage of participant selection. All the study participants were entirely voluntary and were provided with written informed consent. This project was approved by the Ethics Committee of the Naval Military Medical University.

### Quality control

Besides inclusion criteria, another two quality control approaches were applied to ensure data quality: firstly, only one set of surveys was accepted from the same Internet Protocol (IP) address; Secondly, the average time to complete the survey was 4.74 ± 3.57(s.d.) min and 99.8% subjects completed the questionnaire within 2–15 min. As a result, surveys were not accepted if exceeded a 2–15 min time limit.

### Demographic questionnaire

The first section of the survey required information about gender, age, education and occupation. Participants were also asked if they were quarantined at home.

### History of contact with epidemic area

Subjects who were Wuhan residents, or had been to Wuhan, or had contacted with anyone from Wuhan during the outbreak of COVID-19 were identified as having a history of contact with epidemic area.

### Sleep quality

According to previous findings, certain types of sleep problems were more common across anxiety-related disorders than others^33,34^. As a result, we only selected the most related items from the Pittsburgh Sleep Quality Index (PSQI)^35^ to investigate sleep quality. 4 items form PSQI were used to measure subjects’ sleep quality. The first was the *subjective sleep quality* measuring participants’ satisfaction with sleep. Subject scored 0 if they had very good subjective sleep quality and scored 3 for very bad subjective sleep quality. The second item collected data on *latency onset of sleep*. Scores varied from 0 ~ 3 with 0 standing for having no problems falling asleep and 3 standing for hard to fall asleep for 3 times or more within a week. The third item was the *sleep disturbance* measured by easily waking during the night or too early in the morning. Subjects scored 0 for no occurrence of the problem and scored 3 if they had the problem for 3 times or more within a week. The last item collected data on everyday *sleep duration* and the scores varied form 0 (more than 7 h) to 3 (less than 5 h). Internal consistency reliability (Cronbach’s α) of the four items was 0.77.

### PTSS

PTSD Checklist for DSM-5 (PCL-5) was used to measure PTSS^36^. PCL-5 is a self-report 5-point Likert-Type scale (0 = not at all, and 4 = extremely) with 20 items measuring PTSS of instruction, avoidance, numbing and hyperarousal. The threshold score is 33. In the current study, the total score of PCL-5 was used and higher total scores indicated severer PTSS. The Chinese version of PCL-5 showed excellent consistency and convergent validity in previous studies^37^. Internal consistency (Cronbach’s α) of PCL-5 in the present study was 0.87.

### Data analysis

5 participants were deleted because their response time was below 2 min. Data from 2027 participants were analyzed. Descriptive analyses were conducted to describe the demographic characteristics of the sample, PCL-5 and sleep quality scores. To explore the influence of history of contact with epidemic area on sleep quality and PTSS, Chi-squared tests were applied to analyze differences of sleep quality by epidemic area contact history and independent *t-*test comparing PTSS in participants with and without epidemic area contact history was used. Linear regression was conducted to analyze associations among epidemic area contact history, sleep quality and PTSS. Interaction effects of sleep quality × epidemic area exposure history on PTSS were analyzed using two-way ANOVAs and simple effect analyses.

## Results

### Describe statistical results of participants, PTSS and sleep quality

Participants’ demographic characteristics were reported in Table 1. The average age was 35.47 ± 11.32 (s.d.) years.

**Table 1:** Demographic characteristics of the sample.

Variables	Total sample (N = 2027)
**Sex, n (%)**	
Male	786 (38.8%)
Female	1241 (61.2%)
**Age, n (%)**	
18–29	679 (33.5%)
30–49	1130 (55.7%)
≥ 50	218 (10.8%)
**Education, n (%)**	
High school and below	254 (12.5%)
Undergraduate or college degree	1310 (64.6%)
Postgraduate degree and above	463 (22.8%)
**Occupation, n (%)**	
Student	345 (17.0%)
Employed	1275 (62.9%)
Self-employed	163 (8.0%)
Unemployed and retired	244 (12.0%)
**Administrative division, n (%)**	
Eastern	1086 (53.6%)
Central South	485 (23.9%)
South West	123 (6.1%)
North west	37 (1.8%)
North	126 (6.2%)
North eastern	170 (8.4%)
**In quarantine, n (%)**	
Yes	1797 (88.7%)
No	230 (11.3%)
**History of contact with epidemic area**	
Yes	230 (11.3%)
No	1797 (88.7%)

**Figure 3:**
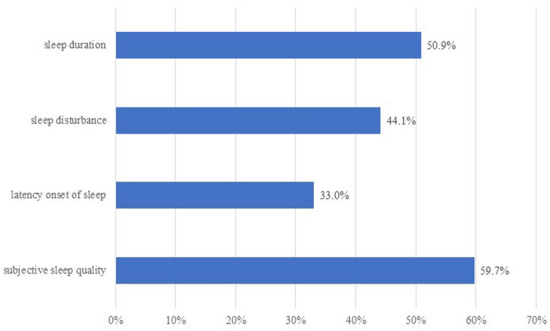
Proportion of participants with sleep quality problems.

Descriptive statistics analysis showed the average score of PCL-5 was 11.77 ± 10.33 (s.d.). For the prevalence of PTSD, 4.7% was positive among the 2027 responders. To calculate participants with sleep quality problems, the scores of the four sleep quality items were recorded into 0.1 value. The original score of 1 ~ 3 was recorded into 1, indicating mild to severe sleep problems and the original score of 0 was recorded into 0, indicating no sleep quality problem. The proportions of participants with sleep quality problems were shown in Fig. 3. As illustrated, unsatisfied subject sleep quality, which influenced nearly 60% participants, was the most common problem, followed by short sleep duration (50.9%), sleep disturbance (44.1%) and sleep onset latency (33.0%).

### Influence of history of contact with epidemic area on sleep quality and PTSS

Subjects with and without epidemic area contact history and their sleep quality and PTSS scores were demonstrated in Table 2. Results illustrated that participants with epidemic area contact history generally showed more problems of latency onset of sleep (χ^2^ = 9.77, P < 0.05) than those not. 36.5% subjects with contact history showed various degrees of sleep onset latency problems compared with 32.6% in subjects without contact history. However, there were no significant differences of other sleep problems between participants with and without contact history. Epidemic area contact history also significantly influenced PCL-5 scores (t_267.38_ = − 2.93, P < 0.05).

**Table 2:** Sleep quality and PCL-5 score by epidemic area contact history.

	Epidemic area contact history				χ^2^	P
	No (n = 1797)		Yes (n = 230)			
	N	%	N	%		
**Subjective sleep quality**						
Very good Good	724	40.3%	92	40.0%	6.643	0.084
	768	42.7%	91	39.6%		
Poor	281	15.6%	39	17.0%		
Very poor	24	1.3%	8	3.5%		
**Latency onset of sleep**						
No	1212	67.4%	146	63.5%	**9.768**	**0.021**
< Once a week	282	15.7%	30	13.0%		
Once or twice a week	218	12.1%	33	14.3%		
≥ Three times a week	85	4.7%	21	9.1%		
**Sleep fragment**						
No	1001	55.7%	132	57.4%	3.996	0.262
< Once a week	286	15.9%	26	11.3%		
Once or twice a week	325	18.1%	43	18.7%		
≥ Three times a week	185	10.3%	29	12.6%		
**Sleep duration**						
> 7 h	872	48.5%	124	53.9%	3.861	0.277
6–7 h	584	32.5%	61	26.5%		
5–6 h	276	15.4%	38	16.5%		
< 5 h	65	3.6%	7	3.0%		
	**Mean**	**s.d**	**Mean**	**s.d**	**t**	**P**
PCL-5 Score	11.49	9.981	14.00	12.546	**− 2.925**	**0.004**

*PCL-5 *PTSD Checklist for DSM-5.

### Influence of history of contact with epidemic area and sleep quality on PTSS

To analyze the influence of epidemic area contact history and sleep quality on PTSS, regression analyses were conducted first. As shown in Table 3, linear regressions with PCL-5 as the dependent variable and epidemic area contact history/sleep quality as independent variables showed that higher scores of epidemic area contact history, subject sleep quality, sleep onset latency, sleep fragment, as well as sleep duration were significantly associated with higher PCL-5 scores, indicating that having contact with epidemic area contact and poor sleep quality were linked to more PTSS.

**Table 3:** Association between contact with epidemic area history, sleep quality and PTSS during outbreak of COVID-19 in China.

Variable	R^2^	AR^2^	B	P	95% CI
**History of contact with epidemic area**					
Yes	0.006	0.005	2.62	< 0.01	(0.94, 4.29)
No			Ref		
**Subjective sleep quality**					
Very good	0.21	0.21	− 25.02**	< 0.001	(− 31.34, − 18.66)
Good			− 21.07**	< 0.001	(− 27.35, − 14.84)
Poor			− 13.30**	< 0.001	(− 19.80, − 6.84)
Very poor			Ref		
**Latency onset of sleep**					
No	0.19	0.19	− 8.59**	< 0.001	(− 12.10, − 5.40)
< Once a week			− 12.25**	< 0.001	(− 15.53, − 9.19)
Once or twice a week			− 17.10**	< 0.001	(− 20.16, − 14.18)
≥ Three times a week			Ref		
**Sleep fragment**					
No	0.16	0.16	− 12.57**	< 0.001	(− 14.63, − 10.63)
< Once a week			− 8.27**	< 0.001	(− 10.51, − 6.10)
Once or twice a week			− 6.27**	< 0.001	(− 8.39, − 4.25)
≥ Three times a week			Ref		
**Sleep duration**					
> 7 h	0.08	0.08	− 12.61**	< 0.001	(− 17.03, − 8.94)
6–7 h			− 10.47**	< 0.001	(− 14.89, − 6.80)
5–6 h			− 6.82**	< 0.001	(− 11.27, − 2.98)
< 5 h			Ref		

**Figure 4:**
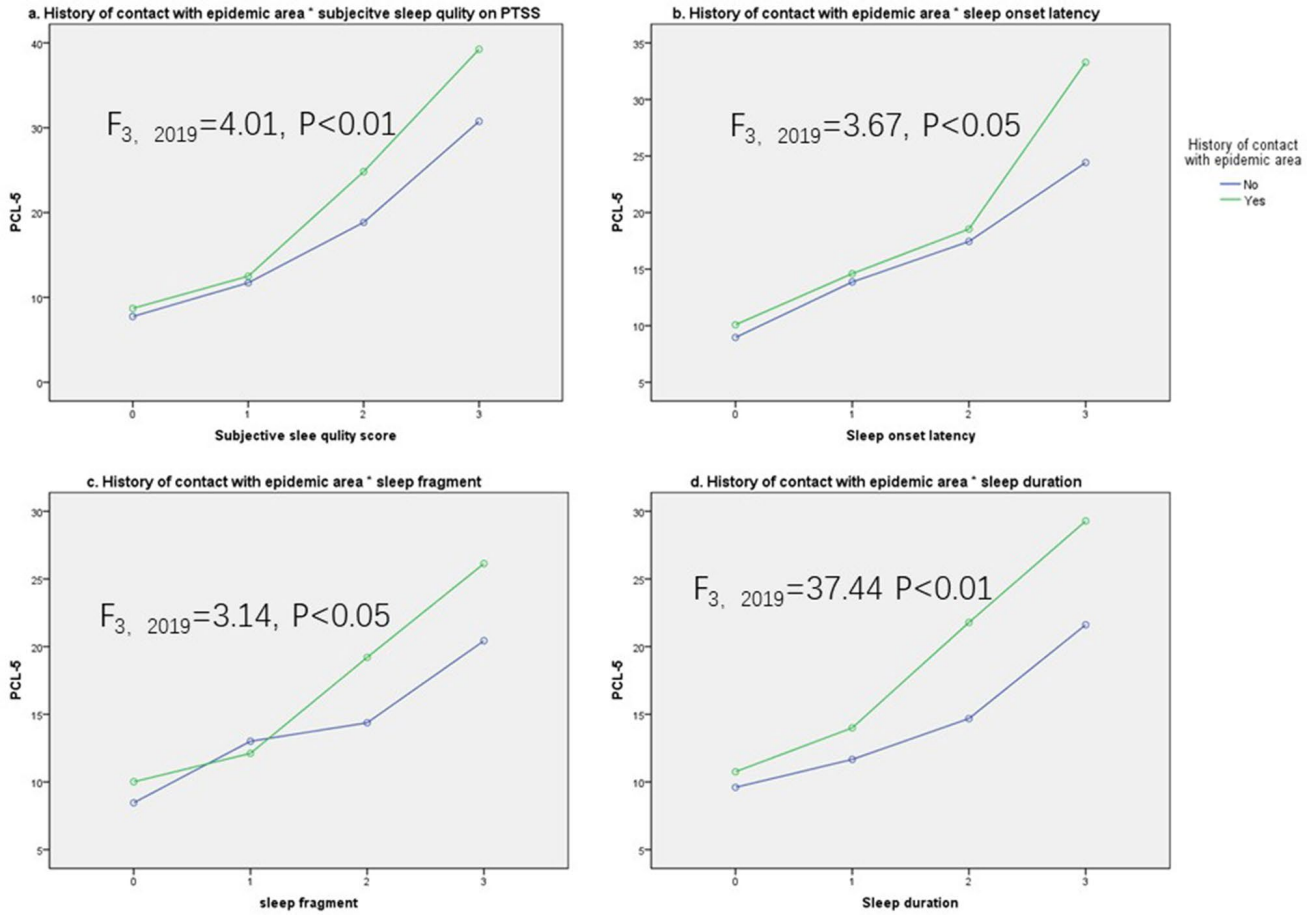
The interaction effect of history of contact with epidemic area × sleep quality on PTSS. *PCL-5 *PTSD Checklist for DSM-5.

To further explore the interaction effect of history of contact with epidemic area × sleep quality on PTSS, the mean values of PCL-5 were entered into four 2 × 4 ANOVAs with exposure history and sleep quality as independent variables. As shown in Fig. 3, results showed a significant interaction effect of contact history × subjective sleep quality on PCL-5 (F_3,2019_ = 4.01, P < 0.01, Fig. 4a). Simple effect tests indicated that there was a significant main effect of contact history on PCL-5 in subjects with relatively poorer subject sleep quality: for subjective sleep quality = 2, the main effect of epidemic contact history was significant, F_1,2019_ = 14.68, P < 0.01; for subject sleep quality = 3, the main effect of epidemic contact history was significant, F_1,2019_ = 5.20, P < 0.05. But in participants with better subjective sleep quality, the main effect of contact history was not significant. Results also demonstrated the interaction effect of contact history × latency onset of sleep (F_3,2019_ = 3.67, P < 0.05, Fig. 4b), but contact history only significantly affected PTSS in those showed the most serious sleep onset latency problems (score 3 points in latency onset of sleep, F_1,2019_ = 15.44, P < 0.01). Interactions of contact history × sleep fragment (F_3,2019_ = 3.14, P < 0.05, Fig. 4c) and contact history × sleep duration (F_3,2019_ = 3.65, P < 0.05, Fig. 4d) were significant. Results of simple effect tests were the same: the main effects of contact history were only significant in subjects who were more easily to wake up and who had shorter sleep duration (scored 3 and 4 points in sleep fragment and in sleep duration, F_1,2019_ = 3.81 ~ 17.27, Ps < 0.05); for participants with less sleep fragment and sleep duration problems, the main effect of epidemic area contact history was not significant. These results indicated that sleep quality regulated the relationship between contact history and PTSS. The main effects of contact history were also significant (F_1,2019_ = 11.76 ~ 15.86, Ps < 0.01). In addition, the four types of sleep problems showed significant main effects on PTSS, F_1,2019_ = 119.89 for subjective sleep quality, F_1,2019_ = 102.24 for sleep onset latency, F_1,2019_ = 72.44 for sleep fragment, and F_1,2019_ = 37.44 for sleep duration, Ps < 0.001.

## Discussion

In the first 2 months of 2020, COVID-19 outbreak in China reached its peak. By the end of our survey, there were more than 20,000 confirmed cases and more than 400 death. Moreover, the confirmed cases and suspected cases were still frantically increasing across China by that time, showing no sign of decline^38^. As Forte et al.^5^ pointed out, the COVID-19 pandemic could be considered as a traumatic event, causing PTSD symptoms and psychological distress. To investigate the potential destructive effects of COVID-19 on mental health, the present study was one of the first to collect data during the highest point of the pandemic to evaluate acute psychological responses and possible risk factors in Chinese. Results revealed that more than 50% participants were not fully satisfied with their sleep quality and at least one out of three participants had various sleep quality problems like sleep onset latency, short sleep duration, and sleep fragment. In a recent study of Italian general population, prevalence rate of poor sleep quality was 57.1% during COVID-19 emergency^24^, also indicating the poor sleep quality during COVID-19 pandemic. Together with this previous study, the present study highlighted that during COVID-19 pandemic, it is very important to keep high-quality sleep. However, the prevalence rate of PTSS in the current study was only 4.7%. Although such number seemed relatively low, it was still within the range of recently reported numbers from 2.7% to 31.8%^39,40^. Sample characterizes, assessing tools and survey time might be reasons behind the differences.

According to National Health Commission of China, until Feb 4, 2020, morbidity and mortality of COVID-19 in Wuhan were much higher above the national level^26^, making exposure to Wuhan a risk factor of infection and a cause of mental health problem. The current study aimed to investigate the influence of potential danger of infection on sleep and PTSS. Results illustrated that contact with epidemic area resulted in more PTSS. This result was in line with previous findings that subjective perception of dangerousness during SARS was related with severity of PTSD symptoms^32^. The result was also in agreement with previous findings that having uncertainty regarding the possibility of contacting the infection, and that the possibility of having had direct contact with people infected by COVID-19 were related with PTSS^12^. Results also highlighted the influence of exposure to epidemic area on latency onset of sleep. Difficulty in falling asleep might be a result of increased pre-sleep cognitions. According to Fast^41^, traumatic experiences caused reactions of fear, increased arousal and re-experience, and these reactions affected pre-sleep cognitions and leaded to chances of sleep onset latency. Increased sleep onset latency then influenced mood, cognition and physiological responses. Interestingly, in another work, only sleep latency and daytime dysfunctions were significantly changed during COVID-19 outbreak compared to general data^24^. Sleep onset latency might be more sensitive to potential threat and higher-level sleep latency was related to more fear reinstatement after trauma expose^42^. Thus, measures to avoid sleep onset latency should be adopted in populations with high infection risk and further studies are needed to explore the unique function of sleep latency in mental health.

To explore the potential influence of epidemic area contact and sleep quality on PTSS, regression and ANOVAs were conducted and the results not only revealed the separate influence of the two factors but also revealed interaction effects. Results showed that subjective sleep quality, delayed sleep onset, sleep disturbance and sleep duration all regulated the effect of epidemic area contact history on PTSS, that is, only those with relatively poor sleep quality were influenced by epidemic area contact history, but participants with good sleep quality would not show severe PTSS even if they had been to Wuhan or had contacted with Wuhan residents. These results indicated that traumatic events provided bases for PTSD, but one’s ability to keep sleep quality might be a protective factor for PTSD. This was line with Germain et al.’s^43^ model of sleep and PTSD which indicated that sleep related more closely with PTSS than trauma events. These results were also consistent with previous studies which showed alters of REM sleep after uncontrolled stress accounted for the long term physiological and behavioral impairments in an animal model^44^. We extended previous findings by showing regulation effect of sleep quality on the relationship between trauma exposure and PTSS in human samples. But it should be noted that further studies are needed, for the present study is a cross-sectional study and no causal conclusion could be drawn. To prevent mental illness and protect mental health during the outbreak of COVID-19 and other infectious diseases, health authorities should be mindful of the influence of infectious risks on psychological reactions, propagate the importance of keeping good sleep quality like ensuring enough sleep time, keeping early hours etc.

There are some limitations that deserve discussion. Firstly, as a result of the cross-sectional design, fluctuations of PTSS and sleep quality during the COVID-19 could not be analyzed. In addition, considering the social distancing requirement, online survey with snowball sampling technic was adopted, which resulted in a biased sample. There were more women, students, and well-educated participants in the present study and interpretation of the results should not ignore this aspect.

## Conclusion

The present study suggested that with rapid spread of COVID-19 in China, 33.0–59.7% Chinese general public showed various sleep quality problems and the prevalence of PTSS reached 4.7% with the self-rating survey. Individuals with and without epidemic contact history showed different levels of PTSS and sleep onset latency under the influence of COVID-19. Epidemic contact history influenced PTSS severity in individuals with poor sleep quality, but not in those with high-quality sleep. These results illustrated the influence of COVID-19 outbreak on mental health in China, and highlighted the importance of keeping good sleep quality in those with emergency exposure experiences.

## Data Availability

The datasets analyzed and materials used in this study are available from the corresponding author (LW) on reasonable request.
